# Treatment outcomes and the role of surgery in gestational trophoblastic neoplasia: a population-based cohort study

**DOI:** 10.2340/1651-226X.2025.43274

**Published:** 2025-06-23

**Authors:** Agnes Larsson, Emelie Wallin, Mats Nilsson, Ulrika Joneborg

**Affiliations:** a Department of Obstetrics and Gynecology, Södertälje Hospital, Stockholm, Sweden; b Department of Oncology and Pathology, Karolinska Institutet/University Hospital, Stockholm, Sweden; c Department of Pelvic Cancer, Karolinska Institutet/University Hospital, Stockholm, Sweden; d Department of Medical and Health Sciences, Linköping University, Linköping, Sweden; e Department of Women’s and Children’s Health, Karolinska Institutet/University Hospital, Stockholm, Sweden

**Keywords:** gestational trophoblastic disease, surgical treatment, choriocarcinoma, epithelioid trophoblastic tumour, placental site trophoblastic tumour

## Abstract

**Background and purpose:**

Cure rates of gestational trophoblastic neoplasia (GTN) are excellent, however the surgical interventions in disease management are not well described. The primary aim of this study was to investigate the incidence and types of surgical procedures used for management of GTN and to report treatment outcomes in a population-based cohort. The secondary aim was to assess the impact of hysterectomy on time to human chorionic gonadotropin (hCG)-normalisation in low-risk GTN.

**Material and methods:**

Medical records of all patients treated for GTN at Karolinska University Hospital, Stockholm, Sweden between 1994 and 2020 were screened for treatment outcomes, types of surgical procedures and complications. Regression models were used to assess if hysterectomy affected time to complete remission in low-risk GTN.

**Results and interpretation:**

Over the 27-year study period, 185 patients with GTN were included. The primary complete remission rate was 98.4% and relapse rate 3.2%. Sixty-four patients (34.6%) underwent at least one surgical procedure; 39/154 (25.3%) of low-risk patients, 17/23 (73.9%) of high-risk patients and all (100%) patients with placental site or epithelioid trophoblastic tumour. No severe complications (Clavien-Dindo ≥3) were observed. Seven of 74 procedures (9.5%) were complicated by bleeding >1,000 mL or surgical site infection. Therapeutic hysterectomy significantly shortened time to hCG-normalisation in the low-risk group (48 vs 74 days, *p* = 0.002).

This population-based study confirms the excellent cure rates and low relapse rates for GTN. Surgery plays an important role in the management of GTN with low risk of complications. Hysterectomy shortens time to hCG normalisation.

## Background

Gestational trophoblastic disease (GTD) is a rare pregnancy-related condition, which originates from the placental trophoblasts. The term GTD includes premalignant entities such as complete and partial hydatidiform moles, as well as malignant variants collectively referred to as gestational trophoblastic neoplasia (GTN). The malignant forms include invasive mole, choriocarcinoma, placental site trophoblastic tumour (PSTT) and epithelioid trophoblastic tumour (ETT) [1]. GTN most commonly derive from molar pregnancies with abnormal trends in human chorionic gonadotropin (hCG) but choriocarcinoma, PSTT and ETT can also arise after any non-molar pregnancy [2,3].

The prognosis for GTN is excellent due to the general sensitivity to chemotherapy with overall cure rates exceeding 90%. The International Federation of Gynaecology and Obstetrics (FIGO) risk scoring system is used for treatment stratification, where patients in the low-risk group will receive single-agent chemotherapy while patients in the high-risk group are treated with more aggressive multi-agent chemotherapy from the outset [4]. The pregnancy hormone hCG acts as an almost perfect tumour marker allowing for easy control of disease progression and treatment response [5]. PSTT and ETT however have a different biological behaviour and generally poorer response to chemotherapy. The FIGO risk scoring system is not applicable for these subgroups and instead the anatomical spread is described according to the FIGO staging system [1].

Although most patients are cured with chemotherapy surgery remains an important part of both diagnosis and treatment of GTN [3]. Few studies, however, report the actual frequency of surgical procedures. Indications for surgical procedures are wide, ranging from diagnostic procedures to acute control of haemorrhage to removal of chemotherapy resistant foci of disease [6]. If the tumour is localised to the uterus, hysterectomy is curative in many cases and can be an option for patients without further pregnancy wish [7,8]. For the less chemotherapy sensitive entities PSTT and ETT, surgery is the mainstay of treatment, also in advanced disease when a combination of surgery and systemic treatment often is necessary to achieve cure [9,10].

In Sweden, diagnosis, and treatment of GTD is centralised to Karolinska University Hospital in Stockholm since July 1, 2020. Even prior to centralisation of care, GTD patients from almost half of the Swedish population were referred to the Karolinska GTD centre for treatment. In the Swedish national guidelines for GTD the indications for surgery are acute procedures such as control of haemorrhage and elective treatment of isolated chemo-resistant tumour lesions, treatment of PSTT and ETT or hysterectomy to reduce the need of chemotherapy in patients not desiring future pregnancies. In accordance with the international literature, second curettage is not routinely performed but can be considered in selected cases [11].

The primary aim of this study was to investigate the incidence and types of surgical procedures in the diagnosis and treatment of GTN and to report treatment outcomes in a population-based setting. The secondary aim was to assess if hysterectomy influences time to hCG-normalisation in low-risk GTN.

## Material and methods

This study was an observational cohort study including patients with GTN in a population-based setting with centralised cancer care. Data from medical records of all patients treated for GTN at Karolinska University Hospital between 1994 and 2020 were included.

### Setting

Treatment for all forms of GTN is publicly available for all residents of Sweden. No privately funded cancer care is available. During the study period, there were five centres in Sweden treating GTN. Karolinska University Hospital was the referral centre for all women with GTN from the Stockholm region and Mid-region of Sweden, an area of currently 4.5 million inhabitants, making up almost half of the total population of 10 million.

### Patients

All Swedish residents are assigned an individually unique national registration number at birth or first permanent residency [12]. This identification number facilitates data collection and patient follow-up and enables linkage between different registries. In this study, all women with a primary diagnosis of GTN treated at Karolinska University Hospital between 1994 and 2020 were included. Patient identification data were retrieved from three different registries: The Karolinska University Hospital local registry; the Regional Cancer Registry and the regional pathology database SymPathy (Tieto AB, Malmö, Sweden). These registries were screened for the International Classification of Diseases (ICD-10), topography and morphology ICD-O/2 and ICD-O/3 and the Systematized Nomenclature of Medicine (SNOMED) codes for complete hydatidiform mole: O01.0, 91000/b, m91000; partial hydatidiform mole: O01.1, 91000/b, 91030/b, m91010, m91030; non-specified hydatidiform mole: O01.9, 91000/b, m91000; invasive mole: D29.2A, 91002, 91001, m91002, m91001; suspected choriocarcinoma: D39.2B; PSTT: D39.2C, 91043/b, 91041/b, m91043; unknown tumour of the placenta: D39.2X; ETT: 91043, 91053, m91053 and choriocarcinoma: C58.9, 91003, m91003, respectively.

Post-molar GTN without a histological diagnosis of malignancy was diagnosed in accordance with the FIGO diagnostic criteria (a plateau of hCG for four measurements over a period of 3 weeks or longer; a rise of hCG for three consecutive weekly measurements over a period of 2 weeks or longer; histological diagnosis of choriocarcinoma) [3]. Women with a non-molar GTN without a histological diagnosis were diagnosed based on increased hCG-levels, typical radiological tumour dissemination and relatively close proximity to pregnancy.

After a diagnosis of GTN, all patients underwent routine examinations in order to assign a risk score. The examinations included serum hCG analysis, gynaecological pelvic examination, pelvic grayscale and power Doppler ultrasound and a chest X-ray. Patients with pulmonary metastases on chest X-ray and/or high-risk disease also had a computerised tomography (CT) of the chest and abdomen and magnetic resonance imaging (MRI) of the brain. Based on the examination results, patients were stratified to receive treatment using the FIGO prognostic scoring system [13]. Patients with a risk score of 6 or below were considered as low risk of treatment failure and received monotherapy, while patients with a risk score of 7 or above were considered as high risk of failure to first-line monotherapy and were given combination chemotherapy from the outset. Patients with PSTT and ETT were not risk scored.

Complete remission was defined as date of hCG normalisation during treatment with persisting normal levels at cessation of consolidation therapy.

The definition of disease relapse currently used is in accordance with the clinical guidelines from the European Organisation for the Treatment of Trophoblastic Disease (EOTTD) (rise of hCG after normalisation and cessation of treatment, including consolidation; two sequential rising hCG values above the reference level of GTD, taken at least 1 week apart; new pregnancy ruled out) [14]. This definition may however have been used inconsequently during the study period, why relapse in this study is defined as persistently increasing hCG levels after the end of treatment with or without clinical or radiological evidence of disease progression. Time to last patient follow-up was December 31, 2023.

### Surgical procedures

The surgical procedures included in this study were any invasive procedure performed after and related to the diagnosis of GTN. Hence, the first curettages performed for diagnosis of hydatidiform moles are not included. The indications for surgery were divided into acute (a non-elective procedure performed due to severe and life-threatening symptoms); diagnostic (a procedure performed to diagnose a condition) and therapeutic (a procedure performed as part of treatment of the disease).

### Complications

Complications screened for were high grade complications corresponding to Clavien-Dindo ≥3 within 30 days of surgery [15]. In addition, bleeding >1,000 mL and surgical site infection, indicating more serious adverse effects of surgery, which could potentially cause subsequent treatment delay, were chosen as additional complication variables. A treatment delay was defined as start of chemotherapy >14 days post-surgery. This time interval was chosen since surgery was considered to replace one cycle of chemotherapy and the cycle interval is 14 days.

### Outcome variable in the low-risk GTN group

Time from start of first treatment, chemotherapy or surgery, to hCG normalisation in days.

Explanatory variables in the low-risk GTN group

The predictor variable investigated was therapeutic hysterectomy.

Chosen confounding variables included the individual variables in the FIGO 2000 risk scoring system; age (under and over 40 years); type of antecedent pregnancy (mole, abortion, full term); interval from antecedent pregnancy (<4, 4–6, 7–12, >12 months); pre-treatment hCG level (<103, 103–<104, 104–<105, ≥105 IU/L); tumour size (<3, 3–<5, ≥5 cm); site of metastasis (lung, spleen-kidney, gastrointestinal, liver-brain); number of metastases (0, 1–4, 5–8, >8); previous chemotherapy failure (none, 1 drug, 2 or more drugs) and total risk score sum (0–6).

### Statistics

Results for different subgroups of GTN are presented as numbers and proportions, means, range, medians, and interquartile range as appropriate. Differences between treatment groups and categorical data were analysed by Fisher’s exact test. The effect of hysterectomy and other explanatory variables on time to hCG normalisation was estimated using linear regression models. The univariable linear model estimated the effect of hysterectomy and other single explanatory variables one at the time on time to hCG normalisation. In the multivariable linear model, explanatory variables that had a statistically significant effect on time to hCG normalisation were analysed. Results from the regression analysis are presented as estimated parameters, 95% confidence intervals and adjusted R-square as appropriate. The significance level was set to 5% and all reported p-values are two-sided. Time to hCG normalisation (time to event) was analysed by *t*-test and Kaplan–Meier statistics stratified on surgery and chemotherapy alone.

### Ethical approvement

This study was approved by the Swedish ethical review authority, DNR 2020-01357, date 2020-06-11.

## Results

**Figure 1:**
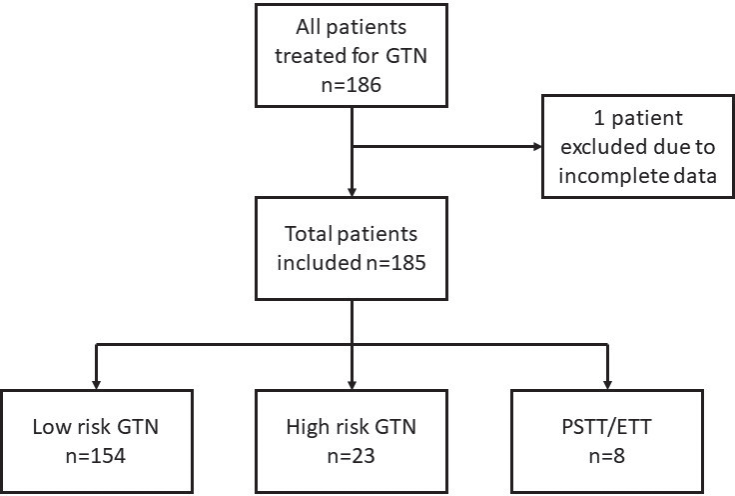
Flowchart of included patients.

During the 27-year study period a total of 186 patients were treated for GTN. One patient was excluded due to incomplete data, leaving 185 patients for further analyses. Two patients were lost to follow-up due to emigration. The flowchart of included patients is presented in Figure 1.

The demographics and primary treatment data are described in Table 1. Of the 185 patients studied, 182 (98.4%) ended primary treatment in complete remission. In the low-risk group 64.0% were cured after first line chemotherapy and 99.4% after treatment with second- and third-line chemotherapy. Eight patients were cured by surgery alone. One patient in the high-risk group and one patient in the PSTT/ETT-group died of treatment resistant disease and one patient in the low-risk group died of unrelated causes during treatment. Two patients, one in the low-risk group and one patient in the PSTT/ETT-group, died from relapsed disease.

**Table 1:** Demographic and treatment data.

	Low risk *n* = 154	High risk *n* = 23	PSTT/ETT *n* = 8
Age in years, mean (range)	34.9 (18.1–55.5)	38.5 (26.4–53.6)	32.8 (18.7–39.6)
hCG IU/L at start of treatment median (IQR), mean (range)	7,039 (950–20,643) 19,974 (17–207,379)	264,023 (60,701–523,377) 412,707 (1,268–1,624,959)	199 (5.5–1,023) 503 (<5–1,262)
Parity *n* (range)	1 (0–5)	2 (0–5)	2 (0–4)
Risk score, mean (range)	2.4 (0–6)	11.9 (7–17)	0
Diagnosis, *n* (%)			
Postmolar GTN	142 (92.2)	3 (13)	
Choriocarcinoma	12 (7.8)	20 (87)	
PSTT			7 (87.5)
ETT			1 (12.5)
Lines of chemotherapy, *n* (%)			
1^st^ line	150 (97.4)	22 (95.6)	5 (62.5)
2^nd^ line	54 (35)	6 (26)	3 (37.5)
3^rd^ line	11 (7.4)	1 (4.3)	2 (25)
4^th^ line	1 (0.6)	1 (4.3)	2 (25)
Oncologic outcome, *n* (%)			
Complete remission	153 (99.4)	22 (95.7)	7 (75)
Dead of disease	0 (0)	1 (4.3)	1 (12.5)
Relapse	5 (3.5)	0 (0)	1 (12.5)

hCG: human chorionic gonadotropin; IQR: interquartile range; GTN: gestational trophoblastic neoplasia; PSTT: placental site trophoblastic tumour; ETT: epithelioid trophoblastic tumour.

The surgical procedures are presented in Table 2. Sixty-four (34.6%) women underwent 74 surgical procedures (acute, *n* = 14; diagnostic, *n* = 15; therapeutic, *n* = 40; combined diagnostic and therapeutic, *n* = 5) as part of their primary treatment. In the low-risk group, 39 of 154 (25.3%) women had 43 surgical procedures compared to 17 of 23 (73.9%) women in the high-risk group with 20 procedures. In the PSTT/ETT-group all eight women underwent at least one surgical procedure.

**Table 2:** Surgical procedures in gestational trophoblastic neoplasia.

	Low risk *n* = 154	High risk *n* = 23	PSTT/ETT *n* = 8	Total *n* = 185
Total women with surgical procedures in primary disease, *n* (%)	39 (25.3)	17 (73.9)	8 (100)	64 (34.6)
Total surgical procedures in primary disease, *n* (%)	43	20	11	74
Indications				
Acute	8	6	0	14 (18.9)
Diagnostic	7	5	3	15 (23.3)
Therapeutic	27	6	7	40 (54)
Diagnostic and therapeutic	1	3	1	5 (6.8)
Types of procedures				
Curettage	15	8	0	23
Hysteroscopy	2	0	3	5
Hysterectomy	20	6	8	34
Pulmonary wedge resection	4	0	0	4
Evacuation of thoracic haematoma	0	1	0	1
Evacuation of cerebral haematoma	0	2	0	2
Other	2	3	0	5

PSTT: placental site trophoblastic tumour; ETT: epithelioid trophoblastic tumour.

The most common indication for surgery was therapeutic (54%) with hysterectomy as the most common procedure. Among the acute operations the most common was curettage, often due to heavy bleeding.

Five surgical procedures are described in the table as ‘other’ and include two gynaecological examinations under general anaesthesia with biopsy or removal of vaginal metastases, one diagnostic laparoscopy, one unilateral salpingo oophorectomy during a caesarian section and one intracerebral embolisation due to haemorrhage.

The complication rate was 9.5% (7/74), including bleeding >1,000 mL, *n* = 5 and infection, *n* = 2. No patients had complications corresponding to Clavien-Dindo ≥3. One patient had a treatment delay of 14 days due to surgical-site infection requiring intravenous antibiotics and hospital care.

The relapse rate for all women treated for GTN was 3.2% (6/185). Surgical procedures were performed in four of these six women with relapsed disease (hysterectomy, n = 2; pulmonary wedge resection, *n* = 4; pulmonary lobectomy, *n* = 1; colectomy, *n* = 1). No complications and no treatment delays were described after these surgical procedures. The indications were in all cases therapeutic or a combination of diagnostic and therapeutic.

**Figure 2:**
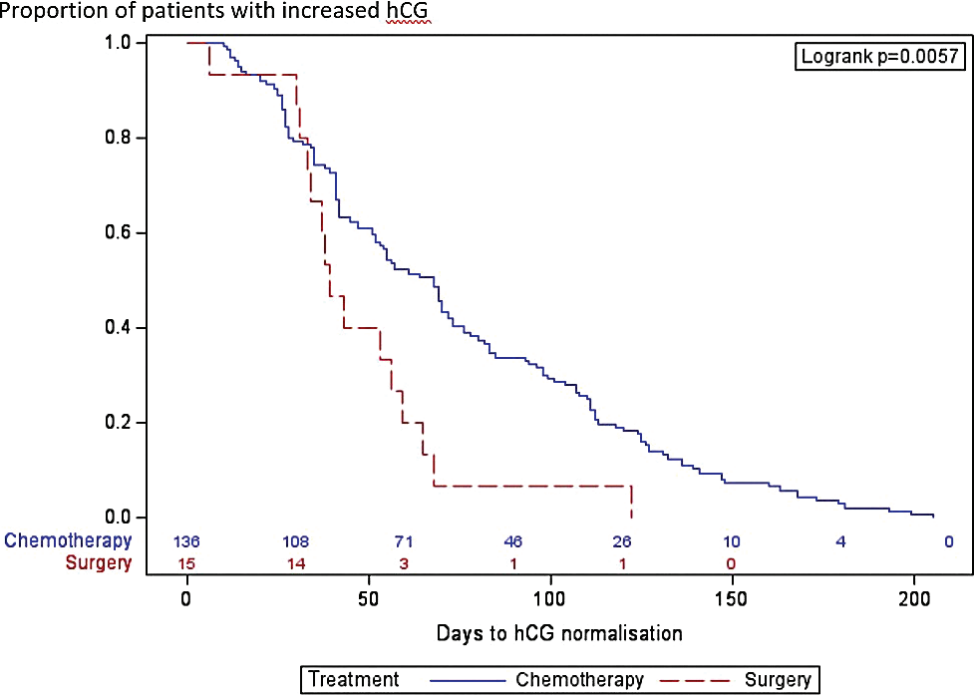
Results from Kaplan–Meier time to event analysis. Event is defined as days to human chorionic gonadotropin (hCG) normalisation, stratified to Chemotherapy (blue line) and Surgery (red line). Logrank test for equality over strata, *p* = 0.0057. Normal hCG levels were reached for 50% in the Chemotherapy group after 68 days and 50% in the Surgery group after 39 days.

Fifteen women in the low-risk group underwent hysterectomy as part of their treatment. Of these, four had hysterectomies upfront, five after one cycle and six after two cycles of single agent Methotrexate. The hysterectomies were performed by laparotomy in 11 patients and by minimally invasive surgery in four patients. Seven patients not completing all items in the FIGO 2000 risk scoring system were omitted from the regression analysis. Among the predefined explanatory variables in the univariable regression analysis, the strongest confounding variables for time to hCG normalisation were hysterectomy, total risk score sum, tumour size and hCG at start of treatment. Location of metastases from the scoring system was excluded in the analysis since all patients had the same value. In the multivariable model, only hysterectomy and hCG at treatment start were retained as predictive factors. There was a statistically significant shorter time to hCG normalisation in the surgery group (47.6 vs. 74.4 days, *p* = 0.002) compared to the chemotherapy alone group as illustrated in Table 3 and Figure 2.

**Table 3:** Background data and time to hCG normalisation by treatment group.

Variables	Chemotherapy *n* = 138	Hysterectomy *n* = 15	*t*-test of difference, *t, df, p*-value
Age (mean, range)	34 (18–56)	47 (27–54)	*-5.9, 151, <0,001*
hCG IU/L at start of treatment mean (range) median (IQR)	20,874 (13–207,379) 7,005 (950–20,643)	20,263 (94–58,000) 13,392 (3,675–34,510)	*0.06, 151, 0.95* *Median test p = 0.4*
Risk score (mean, range)	2 (0–6)	3 (0–6)	*-1.7, 151, 0.09*
Time to hCG normalisation (days) Mean (range)*	74 (10–205)	48 (6–122)	*3.4, 26, 0.002*

* Note that one outlier in the chemotherapy group with 378 days to normalisation is omitted from the analysis

hCG: human chorionic gonadotropin; IQR: interquartile range.

## Discussion

The results from our population-based comprehensive study on outcomes after treatment for GTN demonstrate an excellent overall cure rate of 98.4%. Surgery has a significant role in the management of GTN, with more than one third of all patients having undergone at least one surgical procedure during the course of their disease. In our low-risk GTN population, hysterectomy significantly shortened time to hCG normalisation.

GTN has a well-documented overall cure rate exceeding 90% due to the pronounced chemotherapy sensitivity of trophoblastic tumours [3]. With the introduction of induction chemotherapy for ultra high-risk patients (FIGO risk score >12, brain, liver, or multiple pulmonary metastases), the risk of treatment induced haemorrhage from the vascularised tumours decreased and cure rates further increased [16,17]. The excellent cure rate of GTN is corroborated by our study with a 98.4% overall primary complete remission over a 27-year study period. The results are also comparable with the only other previous Swedish report on treatment outcomes of GTN demonstrating a 96.5% complete remission rate in 58 patients treated at Karolinska University Hospital between 1966 and 1986 [18]. The overall low relapse rate of 3.2% in our study is lower than the 8.6% described in the previous study of the same population but compares to results from a nationwide British study of 4,201 patients reporting an overall relapse rate of 4.7% [19]. A possible reason for the decreased relapse rate in the Swedish population might be introduction of more effective chemotherapy, such as the widely used EMA/CO regimen, over time [20]. With increased centralisation of care of rare diseases in Sweden, it is also possible that the general awareness of GTD has increased and contributed to a more uniform diagnosis and treatment over the years.

The rate of resistance to first line Methotrexate in low-risk disease is reported to 57–90% in the literature but does not affect overall cure rates, which approach 100% [21]. The results of our study are in accordance with these numbers with 99.4% of low-risk patients being cured despite a 35% therapy switch from primary Methotrexate. In our high-risk group, with risk scores ranging between 7 and 17, the vast majority (95.7%) achieved complete remission after combination chemotherapy. Alifrangis et al. described similar results for high-risk GTN, with 94.3% overall survival after exclusion of patients with non-gestational tumours and elimination of early deaths by induction chemotherapy in selected patients [16]. Goldstein et al. also reported treatment outcomes for high-risk GTN divided by stage with high cure rates in stages II and III, but lower rates in stage IV [5].

The results of our study demonstrate that surgery is important in the management of GTN. The frequency of surgical procedures in low-risk GTN, high-risk GTN and PSTT/ETT were approximately 25%, 75% and 100%, respectively. While several studies have examined the frequency of hysterectomy or surgery for chemotherapy-resistant disease, few have reported the overall rate of surgical interventions in GTN. A systematic review by Albright et al. on treatment and outcome of high-risk GTN, including 35 reports from 20 countries, concluded that 35.8% of all patients underwent surgery as part of their treatment. Only eight studies reported surgery in ≥50% of patients [22]. In this review, four of the studies were limited to high-risk patients with metastatic disease and one study to ultra-high-risk disease. The number of surgical procedures for high-risk patients in our series is comparatively high. This may partly reflect the fact that we have included acute procedures after the GTN diagnosis as surgical procedures and partly that patients with GTN were traditionally treated by gynaecological oncologists, with easy access to surgery. Since 2018 GTN is treated by medical oncologists in close collaboration with gynaecological oncologists. In line with the general treatment recommendations, surgery was performed in all patients with PSTT/ETT [8].

Surgical indications were categorised into acute, diagnostic and therapeutic procedures for a more detailed description of the utilisation of surgery. Acute surgeries were in all cases performed to control heavy or life-threatening bleeding. A previous study of the same population between 1966 and 1992 demonstrated that 11% (10/92) of patients with GTN were subjected to acute surgical procedures, all but in once case due to haemorrhage. In seven out of 10 patients, the diagnosis was unknown at the time of surgery [23]. The diagnostic surgeries were done as part of an investigation to reach a diagnosis, for example curettage. Some surgical procedures were given both the indication diagnostic and therapeutic. These procedures were often done to confirm a diagnosis and were at the same time therapeutic as the tumour was removed, i.e. pulmonary wedge resection. The therapeutic procedures were performed to reduce duration of treatment or for chemotherapy resistant foci of disease. These indications and types of surgical procedures are in accordance with those widely described in the literature [8].

Further in line with previous studies, we found an association between hysterectomy and time to hCG normalisation in patients with low-risk GTN [24]. In our cohort, women undergoing a therapeutic hysterectomy had a significantly decreased time to marker normalisation compared to women treated with chemotherapy only, (48 vs 74 days, p = 0.002). As expected, removing the bulk of the tumour induced complete remission in most cases. Seckl et al described that the half-life for hCG is ≤48 hours after surgery if all disease has been removed [11]. Eysbouts et al showed that for patients with localised disease treated with primary hysterectomy, treatment duration was significantly shorter (mean 3.2 weeks and 8.0 weeks, respectively, *p* = 0.01) and number of administered chemotherapy cycles lower than patients in a matched control group treated with chemotherapy only [24]. Hysterectomy as a treatment option for patients without a pregnancy wish is also described in international guidelines [7]. The women with low-risk GTN who underwent therapeutic hysterectomies in our study had no wish to retain fertility. They were well informed of the excellent cure rates with chemotherapy, but actively chose hysterectomy to hopefully shorten duration of treatment. In our setting and during the study period 1994–2020, the standard treatment was to give one to two cycles of Methotrexate with folinic acid rescue before hysterectomy 2 weeks after the last chemotherapy cycle. The length of treatment was therefore at least 3–5 weeks before surgery. This practice was however not completely consistent over the study period, and four out of 15 patients had hysterectomy upfront. Duration of treatment and time to normal hCG levels after elective hysterectomy are therefore not directly comparable with other studies.

One concern regarding surgical procedures for tumours, which might require subsequent chemotherapy is the risk of postoperative complications leading to treatment delays possibly affecting treatment outcomes. GTN is a highly proliferative disease in which prompt initiation of chemotherapy and strictly maintaining treatment intervals is crucial for optimal treatment response. The complication rate after surgery in our cohort was however reassuringly low with no severe complications and only one treatment delay due to surgical site infection.

Strengths of our study include the population-based setting with centralised cancer care, linkage between registries made possible by the individually unique national registration number in Sweden and the complete data set with only one patient being excluded due to incomplete data. The results are representative of the entire region and approximately half of the Swedish population. In rare diagnosis with few diagnosed patients, centralised and population-based care gives us an opportunity to study this patient group.

Among limitations was the slight difference in timing of surgical treatment across the 27-year study period. Time to hCG normalisation obviously differs depending on how many chemotherapy cycles the patient has received before hysterectomy and is not directly comparable to reports from other centres with a different practice. Also, the long inclusion period introduces a time trend bias and indication for surgery or shift of therapy may have changed during this time period.

To conclude, this population-based study confirms the excellent cure rates and low relapse rates of GTN. Although chemotherapy is the mainstay of treatment, surgery plays an important role both for diagnosis and treatment and to manage acute complications with low surgical complication rates. Therapeutic hysterectomy shortens time to complete remission in women who do not wish to retain fertility.

## Authors’ contribution

AL: Data collection, contributed towards data or analysis tools, wrote the article and approved of the final manuscript.

EW: Data collection, conceived and designed the analysis, contributed in data or analysis tools, wrote the article and approved of the final manuscript.

MN: Assisted in data or analysis tools, performed the analysis, wrote the article and approved of the final manuscript.

UJ: Conceived and designed the analysis, performed data collection, and contributed towards data or analysis tools, wrote the article and approved of the final manuscript.

## Disclosure statements

The authors report no competing interests to declare.

## Data availability statement

The data supporting the findings of this study are published. Individual participant data are not publicly available for ethical reasons and restrictions related to data sensitivity.
